# Impact of Prior Kidney Transplantation on Symptom Burden and Health-Related Quality of Life in Incident Dialysis Patients

**DOI:** 10.1016/j.xkme.2026.101357

**Published:** 2026-04-09

**Authors:** Tessa S. Schoot, Thomas S. van Lieshout, Alferso C. Abrahams, Esmee Driehuis, Angèle P.M. Kerckhoffs, Luuk B. Hilbrands, Friedo W. Dekker, Brigit C. van Jaarsveld, Anna A. Bonenkamp, DOMESTICO study group

**Affiliations:** 1 Department of Nephrology, Radboud University Medical Center, Nijmegen, the Netherlands; 2 Department of Nephrology, Amsterdam Cardiovascular Sciences, Amsterdam University Medical Center, Amsterdam, the Netherlands; 3 Department of Nephrology and Hypertension, University Medical Center Utrecht, Utrecht, the Netherlands; 4 Department of Nephrology and Department of Geriatric Medicine, Jeroen Bosch Hospital, ‘s-Hertogenbosch, the Netherlands; 5 Department of Clinical Epidemiology, Leiden University Medical Center, Leiden, the Netherlands; 6 Nephrocare Diapriva Dialysis Center, Amsterdam, the Netherlands

**Keywords:** Dialysis, health-related quality of life, kidney transplant failure, mental health, physical health, symptom burden

## Abstract

**Rationale & Objective:**

Data on symptom burden and health-related quality of life (HRQoL) in patients with kidney graft failure returning to dialysis are limited. This study therefore assessed these parameters during the first year of dialysis in prior-transplant patients and compared them to transplant-naïve incident dialysis patients.

**Study Design:**

Multicenter prospective cohort study: Dutch nOcturnal and hoME dialysis Study To Improve Clinical Outcomes (DOMESTICO).

**Setting & Participants:**

Adult patients who initiated dialysis treatment.

**Exposure:**

Prior kidney transplantation or transplant-naïve status.

**Outcomes:**

Symptom burden (Dialysis Symptom Index) and HRQoL (Short Form-12) at dialysis initiation and 3, 6, and 12 months later.

**Analytical Approach:**

Linear mixed models, adjusted for confounders such as age and comorbid conditions.

**Results:**

A total of 161 prior-transplant and 1,475 transplant-naïve patients were included. Symptom burden did not differ between prior-transplant and transplant-naïve patients (mean differences during first year of dialysis treatment: number of symptoms, 0.1 [95% CI, −0.6 to 0.8]; overall symptom severity score, 0.8 [95% CI, −1.5 to 3.0]). However, prior-transplant patients had lower HRQoL scores at dialysis initiation (physical, −2.6 [95% CI, −4.2 to −0.9]; mental, −2.0 [95% CI, −3.7 to −0.3]), which persisted during the first year of dialysis for physical HRQoL (mean difference in first year, −2.1; 95% CI, −3.3 to −0.8). Mental HRQoL was no longer different between prior-transplant and transplant-naïve patients (−0.8; 95% CI, −1.9 to 0.4).

**Limitations:**

Detailed data regarding the kidney transplantations were not available. Data on psychosocial counseling and guidance were not collected.

**Conclusions:**

We observed lower HRQoL in prior-transplant compared with transplant-naïve patients at dialysis initiation, which may be related to graft rejection and an immunosuppressed state. These findings suggest that patients with graft loss could benefit from additional support to address physical and mental well-being during the transition to dialysis treatment.

## Plain-language Summary

This study aimed to better understand the challenges faced by kidney transplant patients who return to dialysis after their graft fails. We compared their symptoms and quality of life with those of patients who have never had a transplant. Our findings show that although both groups had similar symptom burdens, prior-transplant patients reported lower physical and mental health at the start of dialysis. By the end of the first year of dialysis treatment, the mental health no longer differed between the 2 groups, but physical health was still lower in prior-transplant patients. In conclusion, the results of this study suggest that prior-transplant patients may benefit from extra support to help manage both their physical and emotional well-being during the transition to dialysis.

Approximately 25% of kidney transplant recipients experience graft failure within the first 10 years after transplantation.^1^^,^^2^ After graft failure, patients often (re)initiate dialysis treatment. In the Netherlands, 11% of incident dialysis patients have a history of kidney transplantation (KT).^3^

Kidney graft loss can significantly impact patients’ mental and emotional well-being, often leading to feelings of shock, sadness, and anxiety.^4^, ^5^, ^6^, ^7^ Additionally, the medical management of patients with graft failure involves complex decisions regarding immunosuppressive therapy and whether to perform transplant nephrectomy,^8^^,^^9^ in addition to standard kidney failure care. Patients with graft failure who (re)initiate dialysis treatment have higher all-cause and infection-related mortality as well as higher hospitalization rates compared with transplant-naïve incident dialysis patients.^9^, ^10^, ^11^, ^12^, ^13^, ^14^, ^15^, ^16^, ^17^

Given the increased disease burden and emotional impact of graft loss, we hypothesized that prior-transplant incident dialysis patients experience a higher symptom burden and lower health-related quality of life (HRQoL) than transplant-naïve dialysis patients. However, longitudinal data on symptom burden and HRQoL in prior-transplant patients returning to dialysis are scarce.

To address this knowledge gap, this study investigated the trajectories of symptom burden and HRQoL in prior-transplant patients during the first year of dialysis treatment and compared these trajectories with those of transplant-naïve incident dialysis patients.

## Materials and Methods

### Study Design

This study used data from Dutch nOcturnal and hoME dialysis Study To Improve Clinical Outcomes (DOMESTICO), a prospective, observational cohort study with 59 participating dialysis centers in the Netherlands and Belgium. The primary aim of DOMESTICO is to compare HRQoL, clinical outcomes, and costs between home dialysis therapies and in-center hemodialysis.^18^

Participants completed patient-reported outcome measures (PROMs) at baseline (within 4 weeks of initiating dialysis), and at 3, 6, and 12 months after initiation. Patients completed the PROMs either at home or during a dialysis session. Sociodemographic and clinical data, such as estimated glomerular filtration rate (calculated with the Chronic Kidney Disease Epidemiology Collaboration [CKD-EPI] formula^19^), comorbid conditions (classified with the Charlson Comorbidity Index^20^), medication use, survival, and complication rates were collected from electronic patient records.

All participants provided written informed consent. The study was approved by the Medical Ethics Committee of Amsterdam University Medical Center and the institutional review boards of participating centers. The study was conducted in accordance with the Declaration of Helsinki and the Dutch ‘Medical Research Involving Human Subjects’ Act. The Strengthening the Reporting of Observational Studies in Epidemiology (STROBE) guideline for cohort studies was adhered to (see Item S2).^21^

### Study Population

In DOMESTICO, incident dialysis patients aged ≥18 years were included. Exclusion criteria were life expectancy <3 months or an expected KT within 3 months. Inclusion ran from December 2017 until December 2022.

For the current study, we excluded participants with missing KT history and participants who did not complete any of the PROMs in the first year. Sociodemographic and clinical baseline characteristics of excluded patients were documented.

### Patient-Reported Outcomes

The primary outcomes of this study were symptom burden and HRQoL, which were assessed with the Dialysis Symptom Index (DSI) and Short Form (SF)-12 questionnaires, respectively.

The DSI is a 30-item questionnaire that evaluates the presence and severity of physical and emotional symptoms in dialysis patients.^22^^,^^23^ For 30 symptoms, participants indicate whether the symptom is present and, if so, rate its severity on a 5-point scale (1 = ‘not at all bothersome’ to 5 = ‘bothers very much’). The results are summarized into 2 scores: the total number of symptoms (ranging from 0 to 30, with higher scores indicating more symptoms) and the overall symptom severity score (ranging from 0 to 150, with higher scores reflecting greater symptom severity).

The SF-12 is a 12-item questionnaire.^24^ Its results are summarized into a Physical Composite Summary (PCS) score and a Mental Component Summary (MCS) score. The PCS score includes physical functioning (limitations in physical activities due to health), role-physical (problems with work or other daily activities as a result of physical health), bodily pain (limitations due to pain), and general health (perceived personal health). The MCS score comprises vitality (feeling tired or worn out vs feeling full of energy), social functioning (limitations in social activities due to physical or emotional problems), role-emotional (problems with work or other daily activities due to emotional problems), and mental health (feeling nervous or depressed vs feeling peaceful, happy, and calm).^25^ PCS and MCS scores range from 0 to 100, with higher scores indicating better HRQoL. PCS and MCS scores were derived using the recommended norm-based method.^24^ In the general population, mean PCS and MCS scores are 50 with a standard deviation of 10.

### Statistical Analysis

Categorical variables are presented as frequencies with percentages, normally distributed data as mean with standard deviation, and nonnormally distributed data as median with interquartile range. *P* ≤ 0.05 was considered statistically significant. Analyses were performed in SPSS version 29 and R version 4.1.3.

Linear mixed models were used to analyze mean differences in symptom burden (DSI total number of symptoms and overall symptom severity score) and HRQoL (PCS and MCS scores) between prior-transplant and transplant-naïve patients during the first year of dialysis treatment and at each study time point. To adjust for potential confounding, age, sex, and Charlson Comorbidity Index were added to the crude models.

As an exploratory analysis, χ^2^ tests were used to analyze differences in the crude prevalence of individual DSI symptoms between prior-transplant and transplant-naïve patients. Within the subgroups of prior-transplant and transplant-naïve patients, McNemar’s tests were used to analyze differences in symptom prevalence between initiation of dialysis and 12 months later.

Missing data were handled using multiple imputation to reduce potential bias.^26^ A mixed-effects imputation model with a random intercept for each patient was used to account for within-subject correlation in the longitudinal data.^27^ The imputation model included all variables from the analytical model along with auxiliary variables predictive of the outcome or exposure (KT status). Variables with >50% missing data were excluded from imputation. Imputations were performed at the item score level.^28^

## Results

**Figure 1:**
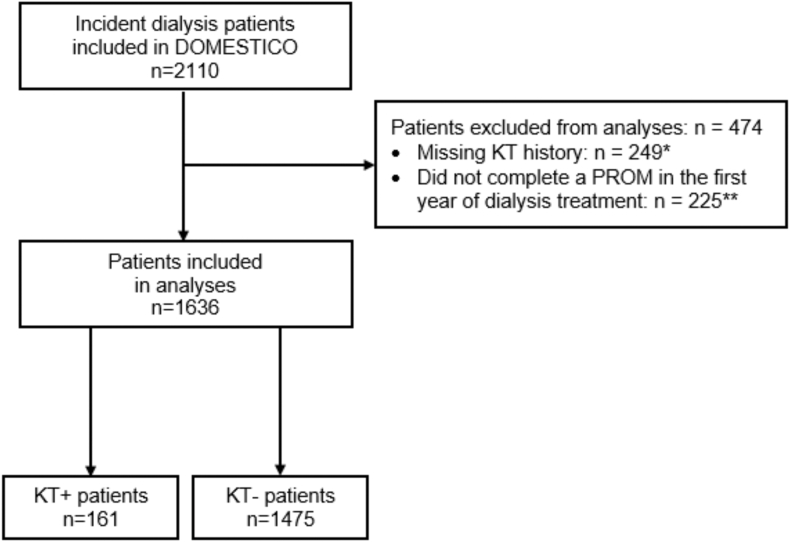
Study population flow chart. Abbreviations: KT, kidney transplantation; KT+ patients, patients with prior KT; KT – patients, patients without prior KT (ie, transplant-naïve patients); PROM, patient-reported outcome measure; SD, standard deviation. ∗ Mean age 63 y (SD 15), 71% men; more characteristics in Table S1. ∗∗ Mean age 62 y (SD 16), 64% men; more characteristics in Table S1.

This study included 1,636 incident dialysis patients: 161 prior-transplant patients and 1,475 transplant-naïve patients (Fig 1). The baseline characteristics of the study population are shown in Table 1. Prior-transplant patients were younger (mean age 55 vs 65 years) and had fewer comorbid conditions and a lower comorbidity score (median Charlson Comorbidity Index 3.0 vs 4.0) than transplant-naïve patients. For prior-transplant patients, mean time since KT was 11.7 years (standard deviation 8.4).

**Table 1:** Baseline characteristics of incident dialysis patients with a history of KT (KT+) and KT-naïve patients (KT−)

	KT+ patients	KT− patients
N	161	1,475
Age (y)	55 (14)	65 (14)
Sex (male)	100 (62%)	974 (66%)
Educational level		
Low	90 (56%)	767 (52%)
Middle/high	48 (30%)	354 (24%)
Unknown	23 (14%)	354 (24%)
Time since KT (y)	11.7 (8.4)	NA
CKD-EPI eGFR at initiation (mL/min/1.73 m^2^)	10.5 (5.1)	9.0 (7.6)
Residual diuresis		
<100 mL/d	2 (1%)	30 (2%)
>100 mL/d	134 (83%)	1136 (77%)
Unknown	26 (16%)	310 (21%)
Unplanned start of dialysis^a^	19 (12%)	251 (17%)
Dialysis modality at start		
HD	103 (64%)	900 (61%)
PD	35 (22%)	295 (20%)
Unknown	23 (14%)	280 (19%)
Vascular access (% of HD patients)		
Arteriovenous fistula	68 (42%)	649 (44%)
Arteriovenous graft	5 (3%)	30 (2%)
Central venous catheter	89 (55%)	782 (53%)
Unknown	0	15 (1%)
Primary kidney disease		
Glomerulonephritis/sclerosis	34 (21%)	192 (13%)
Pyelonephritis	14 (9%)	74 (5%)
Polycystic kidneys	19 (12%)	89 (6%)
Hypertension/renal vascular disease	19 (12%)	413 (28%)
Diabetic kidney disease	16 (10%)	310 (21%)
Other	43 (27%)	280 (19%)
Unknown	14 (9%)	118 (8%)
Charlson Comorbidity Index	3.0 (2.0-4.0)	4.0 (2.0-5.0)
Comorbid conditions		
Diabetes mellitus	42 (26%)	531 (36%)
Cerebrovascular event	2 (1%)	59 (4%)
Peripheral vascular disease	13 (8%)	236 (16%)
Chronic lung disease	8 (5%)	133 (9%)
Malignancy	14 (9%)	221 (15%)

*Note:* Values are n (%), mean (standard deviation), or median (interquartile range).

Abbreviations: eGFR, estimated glomerular filtration rate calculated with the Chronic Kidney Disease Epidemiology Collaboration (CKD-EPI) formula; HD, hemodialysis; KT, kidney transplantation; KT+, prior-transplant patients; KT−, transplant-naïve patients; NA, not applicable; PD, peritoneal dialysis.

a Definition: Patients who initiated dialysis acutely, for example, due to severe hyperkalemia or while being admitted to the Intensive Care Unit.

Baseline characteristics of patients excluded from the analyses were comparable to those of the included transplant-naïve patients (Table S1), except for patients with missing PROMs, who were more likely to have an unplanned start of dialysis, to have a lower estimated glomerular filtration rate, and to use a central venous catheter.

Table S2 shows the percentage of missing data for each question of the SF-12 and DSI at each time point.

### Symptom Burden

**Figure 2:**
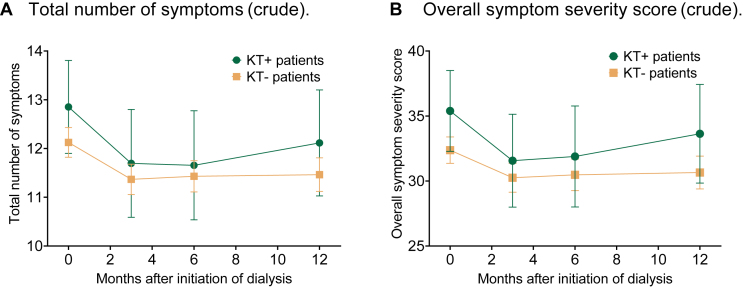
Unadjusted symptom burden (Dialysis Symptom Index summary scores) during the first year of dialysis treatment for patients with (KT+) and without (KT−) prior kidney transplantation. Error bars represent the 95% confidence intervals. (A) Total number of symptoms (crude model; range 0-30, higher scores indicate more symptoms). (B) Overall symptom severity score (crude model; range 0-150, higher scores indicate higher symptom burden). Abbreviations: KT, kidney transplantation; KT+, prior-transplant patients; KT−, transplant-naïve patients.

In both prior-transplant and transplant-naïve patients, the number of symptoms and overall symptom severity score decreased during the first 3 months of dialysis treatment (Fig 2, crude models). In prior-transplant patients, symptom burden increased again between 6 and 12 months after initiation of dialysis. In transplant-naïve patients, symptom burden remained stable between 3 and 12 months after initiation.

**Figure 3:**
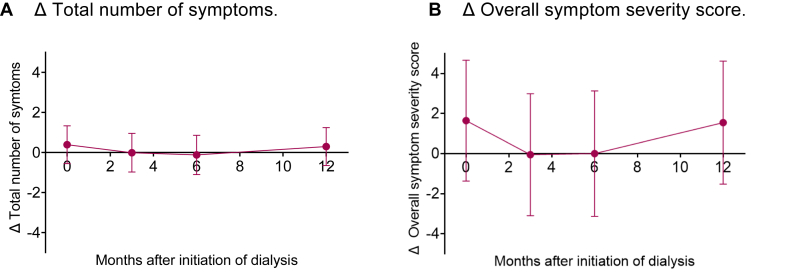
Differences in symptom burden (Dialysis Symptom Index summary scores) between patients with (KT+) and without (KT−) prior kidney transplantation (KT) during the first year of dialysis treatment, adjusted for age, sex, and Charlson Comorbidity Index. Positive values indicate that symptom burden was higher in KT+ patients than in KT− patients. (A) Δ Total number of symptoms. (B) Δ Overall symptom severity score.

After adjustment for confounders, mean symptom burden was not significantly different between prior-transplant and transplant-naïve patients during the first year of dialysis treatment (mean differences in the first year in number of symptoms, 0.1 [95% CI, −0.6 to 0.8], and overall symptom severity score, 0.8 [95% CI, −1.5 to 3.0]; Table 2). At the individual time points (initiation of dialysis and 3, 6, and 12 months later), symptom burden was not significantly different either (Fig 3).

**Table 2:** Differences (with 95% confidence interval) in symptom burden and HRQoL during the first year of dialysis treatment between KT+ and KT− patients.

	Crude model	*P*	Model 1^a^	*P*	Model 2^b^	*P*
Total number of symptoms	0.5 (−0.4 to 1.3)	0.261	0.0 (−0.7 to 0.7)	0.935	0.1 (−0.6 to 0.8)	0.714
Overall symptom severity score	2.2 (−0.8 to 5.1)	0.147	0.4 (−1.9 to 2.7)	0.745	0.8 (−1.5 to 3.0)	0.510
PCS score	−1.0 (−2.7 to 0.6)	0.211	−1.7 (−3.0 to −0.4)	0.008	−2.1 (−3.3 to −0.8)	0.002
MCS score	−1.5 (−3.2 to 0.1)	0.061	−0.7 (−1.8 to 0.5)	0.254	−0.8 (−1.9 to 0.4)	0.177

*Note:* A negative value indicates that scores were lower in patients with a prior KT compared with patients without a prior KT. Higher PCS and MCS scores indicate better HRQoL, and lower Dialysis Symptom Index summary scores indicate lower symptom burden.

Abbreviations: HRQoL, health-related quality of life; KT, kidney transplantation; KT+, prior-transplant patients; KT−, transplant-naïve patients; MCS, Mental Composite Summary; PCS, Physical Composite Summary.

a Model 1: adjusted for age and sex.

b Model 2: adjusted for age, sex, and Charlson Comorbidity Index.

### HRQoL

**Figure 4:**
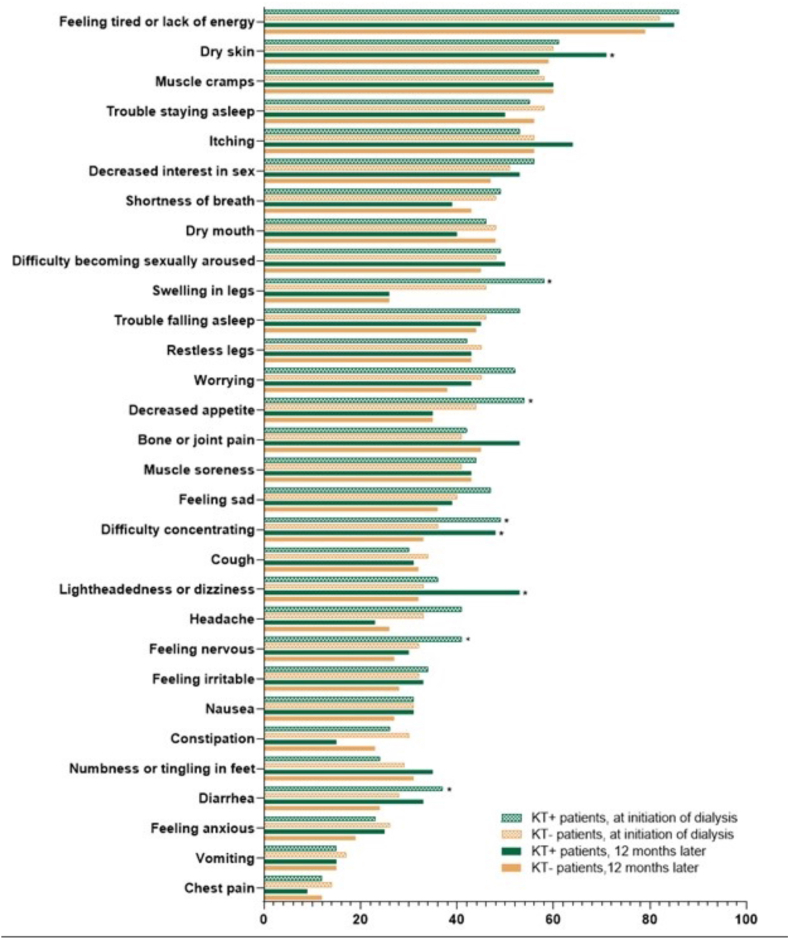
Trajectories of crude physical and mental HRQoL (SF-12) during the first year of dialysis treatment. Error bars represent the 95% confidence intervals. (A) Physical HRQoL (crude model; range 0-100, higher scores indicate better physical HRQoL). (B) Mental HRQoL (crude model; range 0-100, higher scores indicate better mental HRQoL). Abbreviations: HRQoL, health-related quality of life; KT, kidney transplantation, KT+, prior-transplant patients; KT−, transplant-naïve patients; SF-12, Short Form 12.

In both prior-transplant patients and transplant-naïve patients, physical and mental HRQoL increased during the first 3 months of dialysis treatment, with a more prominent improvement in prior-transplant patients (Fig 4, crude models). HRQoL remained stable during the rest of the year in both patient groups.

**Figure 5:**
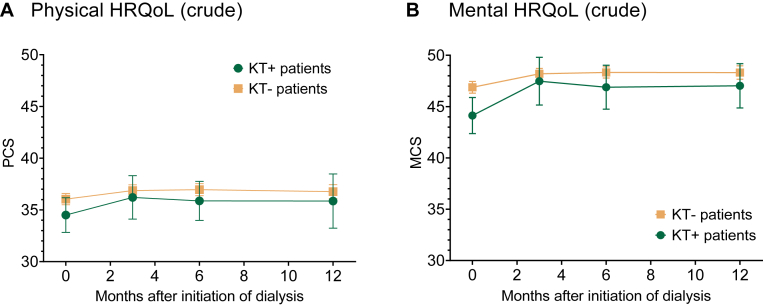
Differences in HRQoL (SF-12) between patients with (KT+) and without (KT−) prior kidney transplantation (KT) during the first year of dialysis treatment, adjusted for age, sex, and Charlson Comorbidity Index. Negative values indicate that HRQoL was lower in KT+ patients compared with KT− patients. (A) Δ PCS. (B) Δ MCS. Abbreviations: HRQoL, health-related quality of life; MCS, mental composite summary; PCS, physical composite summary; SF-12, Short Form 12.

In adjusted analyses, physical HRQoL was significantly lower in prior-transplant patients during the first year of dialysis treatment (mean difference in PCS in the first year, −2.1; 95% CI, −3.3 to −0.8; Table 2). At individual time points, mean physical HRQoL was significantly lower in prior-transplant patients than in transplant-naïve patients at dialysis initiation (difference in mean PCS, −2.6; 95% CI, −4.2 to −0.9), as well as 3 and 6 months later (Fig 5A).

Mental HRQoL, adjusted for confounders, was not significantly different between prior-transplant and transplant-naïve patients during the first year of dialysis treatment (mean difference in MCS in the first year, −0.8; 95% CI, −1.9 to 0.4; Table 2). Although mental HRQoL was significantly lower in prior-transplant patients compared with transplant-naïve patients at initiation of dialysis (difference in mean MCS, −2.0; 95% CI, −3.7 to −0.3), mental HRQoL was no longer significantly different between prior-transplant and transplant-naïve patients 3, 6, and 12 months later (Fig 5B).

### Individual Symptoms (Exploratory Analysis)

**Figure 6:**
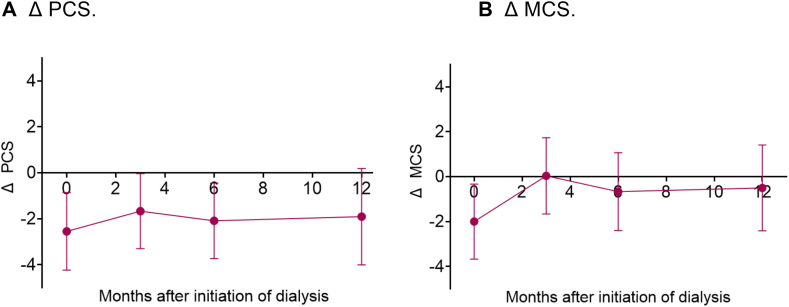
Prevalence of the 30 individual symptoms, assessed by the Dialysis Symptom Index, at initiation of dialysis and after 12 months for prior-transplant (KT+) and transplant-naïve (KT−) patients. Symptoms are listed in order of prevalence (from high to low) at the initiation of dialysis. Asterisks (∗) indicate statistically significant differences between KT+ and KT− patients (χ^2^ test). Abbreviation: KT, kidney transplantation.

The 3 most prevalent symptoms at initiation of dialysis were ‘feeling tired’ (prevalence 86% in prior-transplant patients and 82% in transplant-naïve patients), ‘dry skin’ (61% and 60%, respectively), and ‘muscle cramps’ (57% and 58%, respectively) (Fig 6). Twelve months later, the same 3 symptoms remained the most prevalent among transplant-naïve patients (79%, 59%, and 60%, respectively) (Fig 6). However, for prior-transplant patients, the top 3 symptoms shifted slightly, with feeling tired (85%) and dry skin (71%) being most prevalent, followed by itching (64%) (Fig 6).

At initiation of dialysis, 5 of the 30 symptoms assessed by the DSI were significantly more prevalent in prior-transplant patients than in transplant-naïve patients: ‘swelling in legs,’ ‘decreased appetite,’ ‘difficulty concentrating,’ ‘feeling nervous,’ and ‘diarrhea’ (Fig 6). Twelve months after initiation of dialysis, the prevalence of ‘difficulty concentrating’ was still significantly higher in prior-transplant patients, as were ‘dry skin’ and ‘lightheadedness’ (Fig 6). The prevalence of the other symptoms did not differ significantly between prior-transplant and transplant-naïve patients at 12 months after initiation of dialysis.

During the first year of dialysis treatment, the prevalence of ‘swelling in legs’ and ‘decreased appetite’ significantly decreased in both prior-transplant and transplant-naïve patients, with a more pronounced reduction in prior-transplant patients (‘decreased appetite’: prior-transplant patients 54% at initiation to 35% 12 months later, *P* = 0.017, transplant-naïve patients 44% to 35%, *P* < 0.001; ‘swelling in legs’: prior-transplant patients 58% to 26%, *P* < 0.001, transplant-naïve patients 46% to 26%, *P* < 0.001). In prior-transplant patients, the prevalence of ‘feeling sad’ and ‘headache’ also significantly decreased between dialysis initiation and 12 months later (41% to 23%, *P* = 0.004, and 47% to 39%, *P* = 0.013, respectively). The prevalence of the remaining symptoms did not differ significantly between dialysis initiation and 12 months later.

## Discussion

This study longitudinally investigated symptom burden and HRQoL in patients with graft loss and in transplant-naïve patients initiating dialysis. Symptom burden did not differ between prior-transplant and transplant-naïve patients. However, prior-transplant patients had lower physical and mental HRQoL scores at dialysis initiation, which persisted during the first year of dialysis for physical HRQoL.

These findings suggest that patients with graft loss could benefit from additional support to address physical and mental well-being during the transition to dialysis treatment, especially as HRQoL-related factors are crucial for their patients’ overall satisfaction.^29^, ^30^, ^31^, ^32^ Moreover, previous research indicated that most patients experiencing graft loss are currently not routinely offered psychological support, which some identified as a critical gap in their care.^7^ In clinical practice, this approach may necessitate a multidisciplinary team involving social workers, mental health specialists, and geriatricians for older adults.

Physical HRQoL may be lower in prior-transplant dialysis patients compared to transplant-naïve patients due to the chronic use of immunosuppressive drugs and their side effects, such as more frequent and severe infections.^33^ Additionally, if the kidney graft is not removed, prior-transplant patients are at risk of graft intolerance syndrome, which can present with elevated inflammatory markers, fever, graft swelling, pain over the graft, and/or hematuria.^33^, ^34^, ^35^ The risk of graft intolerance is especially high in the first year of dialysis treatment.^33^^,^^35^ Moreover, prior-transplant patients may require graft nephrectomy, for example, in case of graft intolerance syndrome or to create space for retransplantation, which is a major surgical procedure associated with significant morbidity and mortality.^34^ Finally, prior-transplant patients typically have a longer duration of chronic kidney disease compared with transplant-naïve patients, which may contribute to their poorer physical HRQoL.

Currently, there is no consensus on the management of immunosuppressive therapy for patients with graft failure due to the lack of clear data.^8^^,^^9^^,^^33^ In general, pros for continuation of immunosuppression include prevention of graft intolerance syndrome and prevention of allosensitization, which is relevant for patients who have the prospect of retransplantation. On the other hand, cons for continuation of immunosuppression include increased susceptibility to infections, increased prevalence of malignancies, and cardiovascular complications. Due to the lack of clear data, local practices with regard to the management of immunosuppressive therapy for patients with graft failure may differ.

In our study, overall symptom burden, assessed using the DSI, did not differ significantly between prior-transplant and transplant-naïve patients, despite lower physical HRQoL (SF-12) in prior-transplant patients. This discrepancy may be due to the DSI’s focus on dialysis-specific symptoms, whereas the SF-12 is not disease-specific.^22^^,^^23^^,^^25^ Additionally, HRQoL is influenced by factors beyond symptoms, including functional status and general health perceptions.^36^ Notably, the higher prevalence of certain symptoms in prior-transplant patients at initiation of dialysis and after 12 months compared with transplant-naïve patients may contribute to their lower (physical) HRQoL.

Our study showed that mental HRQoL at initiation of dialysis was lower in prior-transplant patients compared with transplant-naïve patients, which may be related to the emotional impact of graft loss. Previous qualitative research has shown that prior-transplant patients often experience shock, sadness, and anxiety after the loss of their kidney graft (function).^4^, ^5^, ^6^ Additionally, managing graft failure requires complex decisions, such as whether to taper immunosuppressive therapy, which can contribute to emotional distress.^8^^,^^9^^,^^33^ However, our results also revealed that, after starting dialysis, mental HRQoL no longer differed significantly between the 2 groups, suggesting that the emotional impact of kidney graft loss may diminish over time.

Similar longitudinal studies in incident dialysis patients are not available. Two cross-sectional studies have previously investigated HRQoL in prevalent dialysis patients with a prior KT.^15^^,^^37^ First, a study in 12 developed counties worldwide included patients waitlisted for KT (n = 1,856 prior-transplant patients and n = 2,806 transplant-naïve patients). The authors observed that physical HRQoL was lower in prior-transplant dialysis patients than in transplant-naïve dialysis patients (mean difference −2.6; 95% CI, −3.4 to −1.8), whereas mental HRQoL did not significantly differ (0.4; 95% CI, −1.4 to 0.5).^15^ In this study, patients had a mean dialysis vintage of 4 years, whereas our study focused on the first year of dialysis treatment. Interestingly, despite this difference, the observed differences in HRQoL in this study and ours are comparable, suggesting that the lower physical HRQoL in prior-transplant patients may persist in the long term.

In contrast, in a smaller Norwegian study^37^ (n = 50 prior-transplant and n = 251 transplant-naïve patients), physical and mental HRQoL did not differ significantly between prior-transplant and transplant-naïve prevalent dialysis patients. Differences between this study and ours may have arisen from variations in the study population, including differences in perspectives on retransplantation (eg, relatively short waiting lists in Norway compared with other countries). More important, the Norwegian study may have been underpowered to detect significant differences.

Our study has some limitations worth noting. First, we did not collect data on psychosocial counseling and guidance, so we cannot rule out the possibility that the rate of such interventions differed between prior-transplant and transplant-naïve patients or across centers. Nevertheless, the observed differences in HRQoL between prior-transplant and transplant-naïve patients underscore the need for additional attention to mental and physical HRQoL in prior-transplant patients, beyond what is currently provided in standard clinical care. Second, although we conducted multiple statistical tests to compare the prevalence of individual symptoms between groups (n = 60 tests) and within groups (n = 60 tests), we did not correct for multiple testing, eg, with a Bonferroni correction. However, as these analyses were exploratory in nature, we prioritized minimizing the risk of type II errors (failing to detect actual differences) over strictly controlling for type I errors (detecting an effect where no true difference exists). Third, 23% of participants from the DOMESTICO cohort were excluded from the present study due to missing prior-transplant status or missing PROM data. Although most baseline characteristics were similar between included and excluded patients, there were differences in the proportion of patients with acute dialysis initiation and the type of vascular access between the 2 groups, which may have introduced selection bias. Finally, detailed data regarding the KTs were not available (eg, preemptive or non-preemptive, living or deceased donor, immunosuppressive regimens), nor was information on whether patients were (re-)listed for KT. Although such data would have been valuable for conducting subgroup analyses, we doubt whether including this information would have significantly altered our conclusions.

The DOMESTICO data allowed us to analyze the impact of prior KT on symptom burden and HRQoL in a large cohort (n = 1,636), with participants from 59 centers. In our cohort, prior-transplant patients were younger and had fewer comorbid conditions than transplant-naïve patients, similar to the cohorts of 2 studies investigating HRQoL in prevalent dialysis patients with a history of KT.^15^^,^^37^ Additionally, 10% of incident dialysis patients in our study had a prior KT, which aligns with rates observed in other countries (ranging from 1% in Japan to 13% in Spain).^15^

In summary, this study described the trajectories of symptom burden and HRQoL in prior-transplant patients initiating dialysis. The observed lower HRQoL at dialysis initiation in prior-transplant compared with transplant-naïve patients suggests that patients with graft loss may benefit from additional support to address physical and mental well-being during this transition.

## Supplementary Material

Supplementary File (PDF) Item S1-S2, Table S1-S2.
