# Erratum to Impact of Prior Kidney Transplantation on Symptom Burden and Health-Related Quality of Life in Incident Dialysis Patients (Kid Med. 2026;8(6):101357)

**DOI:** 10.1016/j.xkme.2026.101420

**Published:** 2026-06-29

**Authors:** Tessa S. Schoot, Thomas S. van Lieshout, Alferso C. Abrahams, Esmee Driehuis, Angèle P.M. Kerckhoffs, Luuk B. Hilbrands, Friedo W. Dekker, Brigit C. van Jaarsveld, Anna A. Bonenkamp, DOMESTICO study group

**Affiliations:** 1 Department of Nephrology, Radboud University Medical Center, Nijmegen, The Netherlands; 2 Department of Nephrology, Amsterdam Cardiovascular Sciences, Amsterdam University Medical Center, Amsterdam, The Netherlands; 3 Department of Nephrology and Hypertension, University Medical Center Utrecht, Utrecht, The Netherlands; 4 Department of Nephrology and Department of Geriatric Medicine, Jeroen Bosch Hospital, ‘s-Hertogenbosch, The Netherlands; 5 Department of Clinical Epidemiology, Leiden University Medical Center, Leiden, The Netherlands; 6 Nephrocare Diapriva Dialysis Center, Amsterdam, The Netherlands

The publisher regrets that the images for Figures 4, 5, and 6 were not presented in the correct order when the article was published in the Issue in Progress. Originally, the image for Figure 4 was presented as Figure 5, Figure 5 was presented as Figure 6, and Figure 6 was presented as Figure 4. The images have been rearranged accordingly and now appear in the appropriate order in the article.

The publisher would like to apologize for any inconvenience caused.

DOI of original article: 10.1016/j.xkme.2026.101357

