# Supplementary material for: Impact of Prior Kidney Transplantation on Symptom Burden and Health-Related Quality of Life in Incident Dialysis Patients
Source: Kidney Med. 2026 Apr 9;8(6):101357. doi: 10.1016/j.xkme.2026.101357 (PMC13196556; doi:10.1016/j.xkme.2026.101357)
Supplement: Supplementary File (PDF) — Item S1-S2, Table S1-S2. [file mmc1.pdf]

Impact of Prior Kidney Transplantation on Symptom Burden and Health-Related Quality of Life in incident Dialysis Patients

Supplementary Material

Table of Contents:

Page 2	Item S1 – DOMESTICO collaborators
Page 5	Item S2 – STROBE checklist
Page 8	Table S1 – Baseline characteristics of all DOMESTICO participants
Page 10	Table S2 – Missing data

Item S1 – DOMESTICO collaborators

Dialysis center	Investigators
Admiraal de Ruyter Hospital Goes	PB Leurs
Albert Schweitzer Hospital Dordrecht	JB van der Net
Alrijne Hospital Leiderdorp	AM Schrander
Amphia Hospital Breda	TT CNossen
AZ Sint-Jan Brugge	AS De Vriese
Bernhoven Hospital Uden	J Lips
Bravis Hospital Roosendaal	KLW Bunthof; M Eshuis
Canisius-Wilhelmina Hospital Nijmegen	MAGJ ten Dam
Catharina Hospital Eindhoven	CJAM Konings
Deventer Hospital	A van Eck van der Sluijs
Dialysis Center Beverwijk	A Lips
Dialysis Center Groningen	A Özyilmaz
Dianet Dialysis Center Amsterdam	A Neradova
Dianet Dialysis Center Utrecht	FTJ Boereboom
Diapriva Dialysis Center Amsterdam	BC van Jaarsveld
Elisabeth-Twee Steden Hospital Tilburg	C Balemans; S van Esch
Elkerliek Hospital Deurne	CR Susanto
Elyse Clinics Amstelveen	G van Breda
Erasmus Medical Center Rotterdam	D Severs
Flevohospital Almere	AH Boonstra
Franciscus Gasthuis & Vlietland Rotterdam	MAM Verhoeven; RW Nette

Gelderse Vallei Hospital Ede	JM Hofstra
Gelre Hospitals Apeldoorn	YM Vermeeren
Groene Hart Hospital Gouda	DHT IJpelaar
Haaglanden Medical Center The Hague	NH Hommes
Haga Hospital The Hague	M van Buren; H Selten
Isala Hospital Zwolle	SHA Diepeveen
Jeroen Bosch Hospital 's Hertogenbosch	EK Hoogeveen
Jessa Hospital Hasselt	T Cornelis
Laurentius Hospital Roermond	S Boorsma
Leiden University Medical Center	FW Dekker; JI Rotmans
Maasstad Hospital Rotterdam	AM van Alphen
Maastricht University Medical Center	EJR Litjens; MH Hemmelder
Martini Hospital Groningen	WMT Janssen
Maxima Medical Center Veldhoven	A Kuijper; CH Beerenhout
Meander Medical Center Amersfoort	RJ Bosma
Medical Center Leeuwarden	AY Adema; L Bierma
Medical Spectrum Twente Enschede	HS Brink; RMJ Wijering
Noordwest Clinics Alkmaar	EL Penne
OLVG Amsterdam	CWH de Fijter; CEH Siegert
Radboud University Medical Center Nijmegen	WAG van der Meijden
Reinier de Graaf Gasthuis Delft	SJ Huisman
Rijnstate Hospital Arnhem	JC Verhave
Saxenburgh Medical Center Hardenberg	G van Kempen

St. Antonius Hospital. Nieuwegein	WJW Bos
St. Jansdal Hospital Harderwijk	KW Mui; SH Binnenmars
Slingeland Hospital Doetinchem	HHTI Klein
Spaarne Gasthuis Hoofddorp	CE Douma
Tergooi Hospital Hilversum	JD Snoep
Treant Zorggroep Emmen	J Mulder
University Medical Center Groningen	CFM Franssen; A Özyilmaz
University Medical Center Utrecht	AC Abrahams; MC Verhaar
Universitair Ziekenhuis Brussel	K François
University Hospitals Leuven	AH Van Craenenbroeck
VieCuri Medical Center Venlo	AJ Luik
Zaans Medical Center	RJL Klaassen
Ziekenhuisgroep Twente	MMG Dekker-Jansen
ZorgSaam Hospital Terneuzen	AG Weenink
Zuyderland Medical Center Sittard	MME Krekels; N ter Braak

Item S2 – STROBE checklist

Manuscript section	Item	Recommendation	This study
**Title and abstract**	1a	Indicate the study's design with a commonly used term in the title or the abstract.	✔ The subtitle is 'a prospective cohort study'.
	1b	Provide in the abstract an informative and balanced summary of what was done and what was found.	✔
**Introduction**	2	Explain the scientific background and rationale for the investigation being reported	✔
	3	State specific objectives. including any prespecified hypotheses	✔ Last paragraph of introduction.
**Methods**	4	Present key elements of study design early in the paper	✔ Methods > Study design.
	5	Describe the setting. locations. and relevant dates. including periods of recruitment. exposure. follow-up. and data collection	✔ Methods > Study design. and Methods > Study population.
	6a	Give the eligibility criteria. and the sources and methods of selection of participants. Describe methods of follow-up	✔ Methods > Study population.
	7	Clearly define all outcomes. exposures. predictors. potential confounders. and effect modifiers. Give diagnostic criteria. if applicable	✔ Methods > Patient-reported outcomes (PROs) and Methods > Statistical analyses > Trajectory of symptom burden and HRQoL.
	8	For each variable of interest. give sources of data and details of methods of assessment (measurement). Describe comparability of assessment methods if there is more than one group	✔ Methods > Study design and Methods > Patient-reported outcomes (PROs)
	9	Describe any efforts to address potential sources of bias	✔ Methods > Statistical analyses.

	10	Explain how the study size was arrived at	✔ Results > First paragraph.
	11	Explain how quantitative variables were handled in the analyses. If applicable. describe which groupings were chosen and why	✔ Methods > Statistical analyses.
	12a	Describe all statistical methods. including those used to control for confounding	✔ Methods > Statistical analyses.
	12b	Describe any methods used to examine subgroups and interactions	✔ Methods > Statistical analyses.
	12c	Explain how missing data were addressed	✔ Methods > Statistical analyses > Missing data imputation.
	12d	If applicable. explain how loss to follow-up was addressed	Not applicable.
	12e	Describe any sensitivity analyses	✔ Methods > Statistical analyses > Sensitivity analyses
**Results**	13a	Report numbers of individuals at each stage of study—eg numbers potentially eligible. examined for eligibility. confirmed eligible. included in the study. completing follow-up. and analysed	✔ Results > First paragraph and Figure 1.
	13b	Give reasons for non-participation at each stage	✔ Figure 1.
	12c	Consider use of a flow diagram	✔ Figure 1.
	14a	Give characteristics of study participants (eg demographic. clinical. social) and information on exposures and potential confounders	✔ Table 1.
	14b	Indicate number of participants with missing data for each variable of interest	✔ Available upon request.
	14c	Summarise follow-up time (eg. average and total amount)	Not applicable.
	15	Report numbers of outcome events or summary measures over time	✔ Results
	16a	Give unadjusted estimates and. if applicable. confounder-adjusted	✔ Supplemental Table 2.1. Figure 1.

		estimates and their precision (eg. 95% confidence interval). Make clear which confounders were adjusted for and why they were included	Results > Statistical analyses > Trajectory of symptom burden and HRQoL.
	16b	Report category boundaries when continuous variables were categorized	Not applicable.
	16c	If relevant. consider translating estimates of relative risk into absolute risk for a meaningful time period	Not applicable.
	17	Report other analyses done—eg analyses of subgroups and interactions. and sensitivity analyses	✔ Results > Sensitivity analyses.
**Discussion**	18	Summarise key results with reference to study objectives	✔ Discussion first paragraph.
	19	Discuss limitations of the study. taking into account sources of potential bias or imprecision. Discuss both direction and magnitude of any potential bias	✔ Discussion.
	20	Give a cautious overall interpretation of results considering objectives. limitations. multiplicity of analyses. results from similar studies. and other relevant evidence	✔ Discussion.
	21	Discuss the generalisability (external validity) of the study results	✔ Discussion.
**Other information**	22	Give the source of funding and the role of the funders for the present study and. if applicable. for the original study on which the present article is based	✔ Authorship page.

Table S1: Baseline characteristics of all DOMESTICO participants

		Patients included in analyses		Patients excluded from analyses	
		KT+ patients	KT- patients	KT status missing	PROMs missing
N		161	1475	249	227
N with prior KT		161	0	UNK	21
Age (years)		55 (14)	65 (14)	63 (15)	62 (16)
Sex (% male)		62%	66%	71%	64%
Educational level*	Low	56%	52%	38%	1%
	Middle/high	30%	24%	24%	1%
	Unknown	14%	24%	38%	98%
Time since KT (years)		11.7 (8.4)	NA	NA	8.4 (7.1))
CKD-EPI eGFR at initiation (ml/min/1.73m^2)		10.5 (5.1)	9.0 (7.6)	8.6 (4.0)	8.6 (4.0)
Residual diuresis	< 100 ml/day	1%	2%	2%	2%
	> 100 ml/day	83%	77%	50%	58%
	Unknown	16%	21%	48%	40%
Acute start of dialysis*		12%	17%	16%	25%
Dialysis modality	HD	64%	61%	49%	63%
	PD	22%	20%	17%	19%
	Unknown	14%	19%	34%	18%
Vascular access (% of HD patients)	Arteriovenous fistula	42%	44%	40%	35%
	Arteriovenous graft	3%	2%	4%	1%
	Central venous catheter	55%	53%	55%	63%
	Unknown	0%	1%	1%	1%

		Patients included in analyses		Patients excluded from analyses	
		**KT+ patients**	**KT- patients**	**KT status missing**	**PROMs missing**
Primary kidney disease	Glomerulonephritis/sclerosis	21%	13%	UNK	10%
	Pyelonephritis	9%	5%	UNK	5%
	Polycystic kidneys	12%	6%	UNK	4%
	Hypertension / Renal vascular disease	12%	28%	UNK	27%
	Diabetic kidney disease	10%	21%	UNK	25%
	Other	27%	19%	UNK	17%
	Unknown	9%	8%	UNK	12%
Charlson Comorbidity Index		3.0 (2.0; 4.0)	4.0 (2.0; 5.0)	4.0 (2.0; 5.0)	4.0 (2.0; 5.0)
Comorbidities	Diabetes mellitus	26%	36%	32%	42%
	Cerebrovascular event	1%	4%	4%	3%
	Peripheral vascular disease	8%	16%	14%	14%
	Chronic lung disease	5%	9%	7%	4%
	Malignancy	9%	15%	19%	13%

Abbreviations: eGFR = estimated glomerular filtration rate calculated with the CKD-EPI formula; ESKD = end-stage kidney disease; KT = kidney transplantation; KT+ patients = patients with prior KT; KT- patients = patients without prior KT; NA = not applicable; UNK = unknown.

Table S2: Missing data

Percentage of missing data for each question of the SF-12 and DSI at each time point.

	T=0	T=3 months	T=6 months	T=12 months
**SF-12**				
Question 1	26.5	34.5	34.5	48.0
Question 2	26.8	35.2	34.5	48.1
Question 3	29.2	36.7	37.4	50.7
Question 4	27.4	36.0	35.7	48.8
Question 5	28.1	36.9	36.1	49.6
Question 6	28.1	35.9	35.5	49.4
Question 7	28.9	37.5	36.7	49.8
Question 8	27.6	35.3	35.3	49.0
Question 9	27.6	35.3	35.0	48.8
Question 10	28.1	35.6	35.4	49.1

Question 11	27.8	35.4	35.1	48.8
Question 12	27.4	35.5	35.2	49.1
**DSI**				
Question 1	26.5	34.8	34.7	48.3
Question 2	26.3	34.7	34.5	48.5
Question 3	26.5	35.0	34.5	48.4
Question 4	26.6	34.7	34.8	48.3
Question 5	26.4	34.8	34.5	48.3
Question 6	26.3	34.8	34.5	48.3
Question 7	26.4	34.7	34.5	48.5
Question 8	26.4	34.9	34.6	48.3
Question 9	26.4	35.0	34.5	48.3
Question 10	26.5	35.1	34.7	48.3
Question 11	26.6	34.8	34.5	48.5

Question 12	26.5	34.9	34.6	48.5
Question 13	26.6	35.0	34.6	48.5
Question 14	26.6	35.0	34.5	48.4
Question 15	26.6	35.0	34.6	48.5
Question 16	26.6	34.8	34.7	48.5
Question 17	27.3	35.1	34.9	48.8
Question 18	27.3	35.3	35.3	48.6
Question 19	27.3	35.3	35.2	48.7
Question 20	27.3	35.2	35.0	48.7
Question 21	27.3	35.4	35.1	48.6
Question 22	27.8	35.3	35.1	48.7
Question 23	27.6	35.5	35.2	48.7
Question 24	27.3	35.2	35.0	48.6
Question 25	27.3	35.2	35.0	48.7

Question 26	27.4	35.3	35.2	48.9
Question 27	27.3	35.6	35.2	48.7
Question 28	27.6	35.4	35.3	48.8
Question 29	31.4	39.0	38.6	52.4
Question 30	31.5	39.4	39.3	53.1
